# Malnutrition in mother-child dyads in the *Brazilian National Survey on Child Nutrition* (ENANI-2019)

**DOI:** 10.1590/0102-311XEN085622

**Published:** 2023-09-25

**Authors:** Dayana Rodrigues Farias, Luiz Antonio dos Anjos, Maiara Brusco de Freitas, Talita Lelis Berti, Pedro Gomes Andrade, Nadya Helena Alves-Santos, Maria Alvim Leite, Carlos Eduardo Raymundo, Elisa Maria de Aquino Lacerda, Cristiano Siqueira Boccolini, Inês Rugani Ribeiro de Castro, Gilberto Kac, Brazilian Consortium on Child Nutrition, Letícia B. Vertulli Carneiro

**Affiliations:** 1 Instituto de Nutrição Josué de Castro, Universidade Federal do Rio de Janeiro, Rio de Janeiro, Brasil.; 2 Departamento de Nutrição Social, Universidade Federal Fluminense, Niterói, Brasil.; 3 Instituto de Estudos em Saúde e Biológicas, Universidade Federal do Sul e Sudeste do Pará, Belém, Brasil.; 4 Faculdade de Medicina, Universidade de São Paulo, São Paulo, Brasil.; 5 Instituto de Estudos em Saúde Coletiva, Universidade Federal do Rio de Janeiro, Rio de Janeiro, Brasil.; 6 Instituto de Comunicação e Informação Científica e Tecnológica em Saúde, Fundação Oswaldo Cruz, Rio de Janeiro, Brasil.; 7 Instituto de Nutrição, Universidade do Estado do Rio de Janeiro, Rio de Janeiro, Brasil.

**Keywords:** Child Nutrition Disorders, Overweight, Growth Disorders, Transtornos da Nutrição Infantil, Sobrepeso, Transtornos do Crescimento, Trastornos de la Nutrición del Niño, Sobrepeso, Trastornos del Crecimiento

## Abstract

Malnutrition affects billions of individuals worldwide and represents a global health challenge. This study aimed to determine the prevalence of malnutrition (undernutrition or overweight) among mother-child dyads in children under 5 years old in Brazil in 2019 and to estimate changes in this prevalence from 2006 to 2019. Individual-level data from the *Brazilian National Survey on Child Nutrition* (ENANI-2019) and the *Brazilian National Survey of Demography and Health of Women and Children* carried out in 2006 (PNDS 2006) were analyzed. Malnutrition outcomes in mother-child dyads included overweight mother and child, undernourished mother and child, and the double burden of malnutrition, i.e., overweight mother and child having any form of undernourishment (stunting, wasting, or underweight). Prevalence and 95% confidence intervals (95%CI) were estimated. Most women (58.2%) and 9.7% of the children were overweight, 6.9% were stunted, and 3.1% of mothers and 2.9% of the children were underweight. The prevalence of overweight in the mother-child dyad was 7.8% and was statistically higher in Southern Brazil (9.7%; 95%CI: 7.5; 11.9) than in the Central-West (5.4%; 95%CI: 4.3; 6.6). The prevalence of overweight mother and stunted child was 3.5%, with statistically significant difference between the extremes of the mother’s education [0-7 vs. ≥ 12 years, 4.8% (95%CI: 3.2; 6.5) and 2.1%, (95%CI: 1.2; 3.0), respectively]. Overweight in the dyad increased from 5.2% to 7.8%, and the double burden of malnutrition increased from 2.7% to 5.2% since 2006. Malnutrition in Brazilian mother-child dyads seems to be a growing problem, and dyads with lower formal education, higher maternal age, and from the South Region of Brazil were more vulnerable.

## Resumo

A má nutrição afeta bilhões de indivíduos em todo o mundo e representa um desafio de saúde global. Este estudo teve como objetivo determinar a prevalência de má nutrição (desnutrição ou excesso de peso) entre díades mãe-filho em crianças menores de cinco anos no Brasil em 2019 e estimar as mudanças nessa prevalência de 2006 a 2019. Foram analisados dados individuais do *Estudo Nacional de Alimentação e Nutrição Infantil* (ENANI-2019) e da *Pesquisa Nacional de Demografia e Saúde da Criança e da Mulher* realizada em 2006 (PNDS 2006). Os desfechos de má nutrição incluíram mãe e filho com excesso de peso, mãe e filho desnutridos e a dupla carga de má nutrição, ou seja, mãe com excesso de peso e filho com qualquer forma de desnutrição (défict de crescimento, magreza ou baixo peso). Foram estimadas a prevalência e os intervalos de 95% de confiança (IC95%). A maioria das mulheres (58,2%) e 9,7% das crianças estavam acima do peso, 6,9% apresentaram déficit de crescimento e 3,1% das mães e 2,9% das crianças estavam abaixo do peso. A prevalência de excesso de peso na díade mãe-filho foi de 7,8% e foi estatisticamente maior no Sul do Brasil (9,7%; IC95%: 7,5; 11,9) do que no Centro-oeste (5,4%; IC95%: 4,3; 6,6). A prevalência de mãe com sobrepeso e filho com déficit de crescimento foi de 3,5%, com uma diferença estatisticamente significante entre os extremos de escolaridade materna [(0-7 *vs*. ≥ 12 anos de estudo), 4,8% (IC95%: 3,2; 6,5) and 2,1% (IC95%: 1,2; 3,0), respectivamente]. O excesso de peso na díade aumentou de 5,2% para 7,8% e a dupla carga de má nutrição aumentou de 2,7% para 5,2% desde 2006. A má nutrição nas díades mãe-filho brasileiras parece ser um problema crescente, sendo as mais vulneráveis aquelas com menor escolaridade e maior idade materna e residentes na Região Sul do Brasil.

## Resumen

La malnutrición afecta a muchas personas en todo el mundo y representa un desafío para la salud mundial. Este estudio tuvo como objetivo determinar la prevalencia de malnutrición (desnutrición o sobrepeso) entre díadas madre-hijo en niños menores de cinco años en Brasil en 2019 y estimar cambios en esta prevalencia de 2006 a 2019. Se analizaron datos individuales del *Estudio Nacional de Alimentación y Nutrición Infantil* (ENANI-2019) y de la *Encuesta Nacional de Demografía y Salud del Niño y de la Mujer* de 2006 (PNDS 2006). Los resultados de la malnutrición incluyeron a madre e hijo con sobrepeso, madre e hijo desnutridos y la doble carga de mala nutrición, es decir, madre con sobrepeso e hijo con cualquier forma de desnutrición (retardo en el crecimiento, emaciación o bajo peso). Se calcularon prevalencias y los intervalos de 95% de confianza (IC95%). La mayoría de las mujeres (58,2%) y el 9,7% de los niños tenían sobrepeso, el 6,9% de los niños presentaban retraso en el crecimiento, y el 3,1% de las madres y el 2,9% de los niños, bajo peso. La prevalencia de sobrepeso en la díada madre-hijo fue del 7,8%, estadísticamente mayor en el Sur de Brasil (9,7%; IC95%: 7,5; 11,9) que en el Centro-Oeste (5,4%; IC95%: 4,3; 6,6). La prevalencia de madres con sobrepeso y de niños con retraso del crecimiento fue del 3,5%, con una diferencia estadísticamente significativa entre los extremos de nivel educativo de la madre [(0-7 vs. ≥ 12 años de nivel educativo), 4,8% (IC95%: 3,2; 6,5) y 2,1% (IC95%: 1,2; 3,0), respectivamente]. El sobrepeso en la díada tuvo un aumento del 5,2% al 7,8%, y la doble carga de mala nutrición aumentó del 2,7% al 5,2% desde 2006. La malnutrición en la díada madre-hijo brasileña resulta ser un problema creciente, siendo las más vulnerables aquellas con menor escolaridad y mayor edad materna y residentes en la Región Sur de Brasil.

## Introduction

Malnutrition affects billions of individuals worldwide and represents a global health challenge ^1^. Overweight has progressively increased in all age groups, whereas underweight and stunting still generates public health concerns in many regions, including Latin America ^1^ ^,^ ^2^ ^,^ ^3^. Global estimates from 2018 show that 149 million children under 5 years of age were affected by stunting, 49 million by wasting, and 40 million by overweight ^4^. Besides these alarming estimates, overweight in children has increased at higher rates than the decline in the prevalence of underweight ^2^. The World Health Organization (WHO) estimates that 40% of women > 18 years old were overweight in 2016 ^5^.

This new nutritional scenario is characterized as the double burden of malnutrition, in which different forms of malnutrition overlap at the individual, household, or national level. At the household level, double burden of malnutrition is the coexistence of underweight/stunting and overweight in household members ^6^. Most studies examining double burden of malnutrition have focused on mother-child dyads ^7^.

The most frequent form of double burden of malnutrition in low- and middle-income countries is an overweight mother and a stunted child in the same household ^8^ ^,^ ^9^. However, we observed a considerable variation on how double burden of malnutrition occur worldwide. A study conducted with data from 54 low- and middle-income countries from 1991 to 2009 found that the mean prevalence of overweight mothers and stunted children was 3.3%, ranging from 0.5%-16% ^10^. This study also showed that the prevalence of overweight mothers and stunted children has increased over the years.

In Brazil, studies using nationally representative data reported that the prevalence of stunting in children and overweight in their mothers decreased from 2.8% in 1999 ^10^ to 2.2% in 2006 ^3^. However, little is known about the different forms of malnutrition in the mother-child dyad in Brazil since then. The description of the occurrence of many forms of malnutrition may help identify their determinants and the most vulnerable subgroups. Thus, this study aims (1) to estimate the prevalence of different forms of malnutrition (undernutrition or overweight) among mother-child dyads in households with children under 5 years old in Brazil in 2019; (2) to describe the distribution of malnutrition according to socioeconomic and demographic factors and food insecurity in 2019; and (3) to describe changes in the prevalence from 2006 to 2019.

## Materials and methods

### Study design, sampling, and population

This descriptive study used data from the *Brazilian National Survey on Child Nutrition* (ENANI-2019), a population-based household survey with Brazilian children under 5 years of age conducted from 2019 to 2020. The detailed methods information is described by Alves-Santos et al. ^11^. The sample design of ENANI-2019 used stratification and clustering, incorporating two or three selection stages. The primary sampling units were the municipalities or census areas, and the elementary sampling units were the households with at least one child under 5 years old at the time of survey. The ENANI-2019 sample is representative of Brazil’s five macroregions, children’s age groups, and sex ^12^. The research population of our analysis consisted of the biological mother-child dyads with anthropometric data available, and that includes children for whom it was possible to estimate all anthropometric growth indices. For multiparous mothers, all mother-child dyads in the household were included in the analyses.

ENANI-2019 has information on 13,936 dyads. Mother-child dyads in which the mother was pregnant during the anthropometric assessment (n = 61) or had missing information of maternal age were excluded (n = 11). Other exclusion criteria were dyads with missing information on child anthropometric measures (n = 4), those in which it was not possible to estimate Z-score of body mass index (BMI)-for-age (BAZ, preterm infants who had gestational age since conception lower than 189 days; n = 188), and children with anthropometric measures considered as implausible weight-for-height (WHZ; Z-score < 5 or > 5); according to the *WHO Child Growth Standard* (n = 13) ^13^ ^,^ ^14^. The final sample included children < 5 years old and their mothers (ranging from 16-64 years old), comprising 13,659 mother-child dyads.

Moreover, the public dataset of the *Brazilian National Survey of Demography and Health of Women and Children* conducted in 2006 (PNDS 2006) ^15^ was analyzed to assess changes in the prevalence of malnutrition. The research population of PNDS 2006 consists of children < 5 years old and their biological mothers (ranging from 15-49 years old), resulting in 4,131 mother-child dyads.

### Anthropometric measures

All anthropometric measurements (weight, height, and length) were obtained using portable SECA equipment (https://www.seca.com/). Weight (kg) and height (cm) values for biological mothers and children ≥ 2 years old were obtained on a digital platform scale (model 813) and stadiometer (model 213). For children < 2 years old, pediatric scales (model 336) and anthropometers (model 417) were used. All measurements were obtained in duplicate. The information was recorded on a mobile data collection device, which identified implausible anthropometric measurements of children (based on the automatic estimation of the Z-score according to the *WHO Child Growth Standard* ^13^) and required birth date confirmation or repetition of the measurement when necessary.

The anthropometric data collection procedures followed the recommendation of the World Health Organization (WHO)/United Nations Children’s Fund (UNICEF) ^14^ and the Brazilian Ministry of Health ^16^, and all field interviewers were trained to obtain anthropometric measurements. Detailed information on training sessions and the standardization of the anthropometric measurements can be found in Anjos et al. ^17^. Anthropometric data quality was evaluated and missing or implausible data were imputed.

### Outcomes: data from ENANI-2019

Data from the first measure of weight and height of the children were used to estimate Z-scores of height-for-age (HAZ), weight-for-age (WAZ), BAZ, and WHZ according to age and sex ^16^. The Z-scores of the indices were obtained based on the *WHO Child Growth Standard* for children born at term ^13^. For preterm children (< 37 gestational weeks) and who had gestational age since conception (estimated by summing gestational age at birth and the postnatal age in days) ranging from 189-454 days, the WAZ and HAZ were estimated using the Intergrowth-21st Project postnatal growth charts ^18^. The BAZ and WHZ were not estimated for preterm children who were under two years of age during the interview due to the lack of reference charts.

The adult biological mother’s (age ≥ 20 years) anthropometric assessment was based on BMI [estimated by dividing the weight (kg) by the square height (m)] classification, using the WHO cutoffs ^19^. For adolescent mothers (< 20 years), the BAZ was estimated and classified according to WHO reference charts ^16^ ^,^ ^20^. Pregnant mothers were not assessed.

Malnutrition in the mother-child dyads entails the combination of overweight (including obesity), undernutrition, and double burden of malnutrition (Box 1). The wasting and overweight classifications were based on WHZ in children under two years of age and BAZ in older children. Furthermore, the prevalence of both conditions was estimated using the BAZ or WHZ only, regardless of the child’s age.

**Box 1:** Malnutrition classifications.

CHILD	
Stunting	HAZ < -2
Wasting	BAZ < -2 (all ages) WHZ < -2 (all ages) BAZ < -2 (age ≥ 2 years old) or WHZ < -2 (age < 2 years old)
Underweight	WAZ < -2
Overweight	BAZ > 2 (all ages) WHZ > 2 (all ages) BAZ > 2 (age ≥ 2 years old) or WHZ > 2 (age < 2 years old)
MOTHER	
Underweight	Adolescents (age < 20 years old ): BAZ < -2 Adults: BMI < 18.5kg/m²
Overweight	Adolescents (age < 20 years old): BAZ > 1 Adults: BMI ≥ 25.0kg/m²
MOTHER-CHILD DYADS	
Overweight	Overweight mother and child (BAZ) Overweight mother and child (WHZ) Overweight mother and child (BAZ or WHZ)
Undernourished	Underweight mother and child stunting Underweight mother and child wasting (BAZ) Underweight mother and child wasting (WHZ) Underweight mother and child wasting (BAZ or WHZ)
Forms of double burden of malnutrition	Overweight mother and child stunting Overweight mother and child wasting (BAZ) Overweight mother and child wasting (WHZ) Overweight mother and child wasting (BAZ or WHZ) Overweight mother and undernourished child (stunting, wasting, or underweight)

BAZ: body mass index (BMI)-for-age Z-score; HAZ: height-for-age Z-score; WAZ: weight-for-age Z-score; WHZ: weight-for-height Z-score.

### Comparison with data from PNDS 2006

In the PNDS 2006 database, HAZ, WAZ, and WHZ values were available, and BAZ data were estimated using individual-level data. The estimation of anthropometric indices was based on the mean of two weight and height measures of the mothers and children. Prematurity was not considered for the nutritional status classification due to the lack of information on gestational age at birth ^15^.

### Data analysis

The analysis was carried out considering the complex sample design using the *survey* package of the R (http://www.r-project.org) ^21^. The Z-scores of anthropometric indices of children were estimated using the R packages *growthstandards* ^22^ and *anthro* ^23^.

Prevalence, 95% confidence intervals (95%CI), coefficient of variation (CV), and total population (mother-child dyads × 1,000) with malnutrition in mother-child dyads were estimated. The CV is a measure of dispersion, indicative of the heterogeneity of the data and is estimated as the ratio of the standard error and estimated prevalence value.

The most prevalent forms of malnutrition in 2019 [overweight in mother-child dyads, overweight mother and stunted child, and overweight mother and undernourished child (stunting, wasting, or underweight)] were described according to socioeconomic and demographic characteristics: Brazilian macroregions (North, Northeast, Southeast, South, and Central-West); the National Wealth Score (IEN) tertiles ^24^; food insecurity level (food security, mild insecurity, and moderate or severe insecurity), measured using the *Brazilian Food Insecurity Scale* (EBIA) ^25^; type of sewage system (general network or others), age group of the child (< 2, ≥ 2 years old); race/skin color of the child (white, mixed-race, black, Asian descendants, indigenous); maternal schooling level (0-7, 8-10, 11, ≥ 12 years of education); maternal age (< 20, 20-29, 30-39, ≥ 40 years old); and marital status (lives with a partner or not). In the descriptive analyses according to the child’s race/skin color, the results “Asian descendants” and “indigenous” children were omitted due to the low precision of the estimates (CV > 30%).

The prevalence of overweight in the mother-child dyads, overweight mother and stunted child, and overweight mother and undernourished child (stunting, wasting, or underweight) was estimated using data from PNDS 2016 for comparison with ENANI-2019. Prevalence estimates and 95%CI were graphically presented. The lack of overlap in the 95%CI of point estimates was considered a statistically significant difference.

### Ethical considerations

The ENANI-2019 was approved by the Research Ethics Committee of the Clementino Fraga Filho University Hospital of the Federal University of Rio de Janeiro (UFRJ; CAAE n. 89798718.7.0000.5257). Data were collected after a parent or caregiver of the child authorized participation in the study through informed consent form.

## Results

In 2019, 58.2% of Brazilian mothers and 9.7% of Brazilian children were overweight (using WHZ or BAZ), 6.9% of children were stunted, and 3.1% of mothers and 2.9% of children were underweight (data not shown in tables). The prevalence of overweight in the mother-child dyad was 6% using the WHZ and 7.8% when the WHZ or BAZ was used. The prevalence of any form of undernourishment in the mother-child dyad was ≤ 0.4%, with all CV estimates > 30%. The double burden of malnutrition (overweight mother and undernourished child) was found in 5.2% of the mother-child dyads in Brazil (Table 1).

**Table 1:** Prevalence (%) of several forms of malnutrition among mother-child dyads in Brazil. *Brazilian National Survey on Child Nutrition* (ENANI-2019).

Forms of malnutrition	%	95%CI	CV (%) *	Mother-child dyads (x 1,000) **
Overweight				
Overweight mother and child (BAZ)	6.4	5.4; 7.4	8.1	889.2
Overweight mother and child (WHZ)	6.0	5.0; 7.1	8.7	841.1
Overweight mother and child (WHZ or BAZ)	7.8	6.5; 9.1	8.4	1,085.0
Undernourished				
Underweight mother and child stunting	0.3	0.2; 0.5	28.8	48.5
Underweight mother and child wasting (BAZ)	0.3	0.1; 0.5	33.5	43.7
Underweight mother and child wasting (WHZ)	0.4	0.1; 0.6	31.3	51.3
Underweight mother and child wasting (WHZ or BAZ)	0.4	0.1; 0.6	31.0	51.7
Double burden of malnutrition decomposition				
Overweight mother and child stunting	3.5	2.9; 4.2	9.1	491.0
Overweight mother and child wasting (BAZ)	1.5	1.1; 2.0	15.4	213.3
Overweight mother and child wasting (WHZ)	1.4	0.9; 1.9	18.9	195.1
Overweight mother and child wasting (WHZ or BAZ)	2.3	1.6; 3.0	15.9	314.2
Overweight mother and undernourished child (stunting, wasting, or underweight)	5.2	4.3; 6.1	9.0	727.0

95%CI: 95% confidence interval; BAZ: body mass index (BMI)-for-age Z-score; CV: coefficient of variation; WHZ: weight-for-height Z-score.

Note: Overweight mother: BAZ > 1 (adolescents, age < 20 years old) or BMI ≥ 25.0kg/m^2^ (adults, age ≥ 20 years old); Overweight child: BAZ > 2 (all ages); or WHZ > 2 (all ages); or BAZ > 2 (age ≥ 2 years old) and WHZ > 2 (age < 2 years old); Child stunting: height-for-age Z-score (HAZ) < -2; Child wasting: BAZ < -2 (all ages) or WHZ < -2 (all ages) or BAZ < -2 (age ≥ 2 years old) and WHZ < -2 (age < 2 years old). Undernourished child: stunting, wasting (BAZ) < -2 (age ≥ 2 years old) and WHZ < -2 (age < 2 years old), or underweight (weight-for-age Z-score - WAZ < -2).

* CV values higher than 30% show a low estimate precision;

** Mother-child dyads (x 1,000), the cell value x 1,000 represents the total of mother-child dyads estimate in the population with the condition.

The prevalence of overweight in mother-child dyads in Southern Brazil (9.7%) was statistically higher than in the Central-West (5.4%). The prevalence was also higher in children ≥ 2 years old (9.1%) than in younger children (5.9%) (Table 2). The prevalence of overweight mothers and stunted children was 3.5% and higher in dyads with fewer completed years of mother formal education, with a statistically significant difference between the extremes (0-7 vs. ≥ 12 years, 4.8% and 2.1%, respectively) (Table 3). The prevalence of overweight mothers and undernourished children was higher in dyads with mothers with lower formal education and older age, but the differences were not statistically significant (Table 4).

**Table 2:** Prevalence (%) of overweight * among mother-child dyads, according to socioeconomic and demographic characteristics and food security in Brazil. *Brazilian National Survey on Child Nutrition* (ENANI-2019).

Characteristics	%	95%CI	CV (%)	Mother-child dyads (x 1,000) **
Brazilian macroregions				
North	6.0	4.4; 7.7	14.1	91.5
Northeast	7.6	5.5; 9.8	14.4	290.9
Southeast	8.2	5.5; 10.9	16.7	456.4
South	9.7	7.5; 11.9	11.5	183.7
Central-West	5.4	4.3; 6.6	10.9	62.5
National Wealth Score (tertiles)				
1st	7.0	5.9; 8.2	8.2	322.8
2nd	7.9	6.2; 9.6	10.7	371.7
3rd	8.4	5.8; 11.1	16.1	390.5
*Brazilian Food Insecurity Scale* (EBIA)				
Food security	8.6	6.8; 10.5	10.8	624.7
Mild insecurity	6.9	5.7; 8.1	8.7	365.7
Moderate or severe insecurity	6.6	3.2; 10.1	26.3	94.6
Sewage				
General network	8.1	6.6; 9.7	9.6	846.4
Other	6.7	5.0; 8.5	13.2	238.6
Child age (years)				
< 2	5.9	4.7; 7.1	10.3	327.5
≥ 2	9.1	7.4; 10.8	9.6	757.6
Child sex				
Male	8.4	6.6; 10.3	11.1	599.2
Female	7.1	5.8; 8.4	9.4	485.8
Child race/skin color				
White	8.1	5.9; 10.3	13.7	467.8
Mixed-race	7.3	6.1; 8.6	8.5	528.5
Black	8.6	5.4; 11.8	18.8	77.5
Maternal educational level (completed years)				
0-7	8.4	6.0; 10.9	14.8	250.1
8-10	7.4	5.7; 9.1	11.6	219.3
11	7.8	6.5; 9.2	9.0	439.7
≥ 12	7.3	4.2; 10.4	21.5	176.0
Maternal age (years)				
< 20	6.6	3.5; 9.7	23.9	61.2
20-29	7.3	6.0; 8.7	9.4	505.2
30-39	8.5	6.5; 10.6	12.2	444.6
≥ 40	8.2	4.9; 11.4	20.4	74.0
Mother lives with partner				
Yes	7.7	6.2; 9.2	9.9	788.6
No	8.1	6.1; 10.0	12.2	296.4

95%CI: 95% confidence interval; CV: coefficient of variation.

* Child overweight was classified using body mass index (BMI)-for-age Z-score (BAZ) > 2 (age ≥ 2 years old) or weight-for-height Z-score (WHZ) > 2 (age < 2 years old); Mothers overweight was classified using BAZ > 1 (adolescents, age < 20 years old) or BMI ≥ 25.0kg/m^2^ (adults);

** Mother-child dyads (x 1,000), the cell value x 1,000 represents the total of mother-child dyads estimate in population with the condition.

**Table 3:** Prevalence (%) of the double burden of malnutrition among mother-child dyads (overweight mother and stunted child) *, according to socioeconomic and demographic characteristics and food security in Brazil. *Brazilian National Survey on Child Nutrition* (ENANI-2019).

Characteristics	%	95%CI	CV (%)	Mother-child dyads (x 1,000) **
Brazilian macroregions				
North	3.3	1.7; 4.9	24.6	49.8
Northeast	3.5	2.2; 4.7	18.3	132.2
Southeast	3.8	2.7; 4.9	14.7	209.3
South	3.6	1.9; 5.3	24.7	68.0
Central-West	2.7	2.1; 3.4	11.9	31.6
National Wealth Scale (tertiles)				
1st	3.7	2.9; 4.6	11.5	172.1
2nd	4.3	2.8; 5.8	17.4	201.8
3rd	2.5	1.3; 3.8	24.7	117.1
*Brazilian Food Insecurity Scale* (EBIA)				
Food security	3.9	2.9; 4.9	12.8	282.5
Mild insecurity	2.9	2.0; 3.9	15.7	155.7
Moderate or severe insecurity	3.7	2.1; 5.3	21.9	52.8
Sewage				
General network	3.7	3.0; 4.5	10.5	389.6
Other	2.9	1.9; 3.8	17.0	101.4
Child age (years)				
< 2	4.5	3.5; 5.5	10.9	251.4
≥ 2	2.9	2.1; 3.7	14.1	239.6
Child sex				
Male	4.0	3.0; 4.9	12.0	282.0
Female	3.1	2.2; 3.9	13.7	209.0
Child race/skin color				
White	3.0	2.1; 3.9	14.8	173.9
Mixed-race	3.9	2.9; 5.0	13.7	283.2
Black	3.1	1.3; 4.9	29.4	27.7
Maternal educational level (completed years)				
0-7	4.8	3.2; 6.5	17.7	143.9
8-10	3.8	2.5; 5.1	17.8	111.3
11	3.3	2.4; 4.2	14.6	185.2
≥ 12	2.1	1.2; 3.0	22.6	50.6
Maternal age (years)				
< 20	2.2	0.5; 3.8	38.7	20.3
20-29	3.2	2.5; 3.9	11.3	218.5
30-39	4.2	3.1; 5.2	13.0	217.5
≥ 40	3.8	1.6; 6.1	30.0	34.6
Mother lives with partner				
Yes	3.5	2.8; 4.2	10.0	362.4
No	3.5	2.3; 4.7	17.5	128.6

95%CI: 95% confidence interval; CV: coefficient of variation.

* Mothers’ overweight was classified using body mass index (BMI)-for-age Z-score (BAZ) > 1 (adolescents, age < 20 years old) or BMI ≥ 25.0kg/m^2^ (adults); Child stunting was classified using height-for-age Z-score (HAZ) < -2;

** Mother-child dyads (x 1,000), the cell value x 1,000 represents the total of mother-child dyads estimate in the population with the condition.

**Table 4:** Prevalence (%) of the double burden of malnutrition among mother-child dyads (overweight mother and undernourished child) *, according to socioeconomic and demographic characteristics and food security in Brazil. *Brazilian National Survey on Child Nutrition* (ENANI-2019).

Characteristics	%	95%CI	CV (%)	Mother-child dyads (x 1,000) **
Brazilian macroregions				
North	3.8	2.2; 5.4	21.8	57.9
Northeast	5.4	3.8; 7.1	15.6	208.3
Southeast	5.7	3.8; 7.5	16.5	315.8
South	5.0	3.2; 6.8	18.1	94.3
Central-West	4.4	3.0; 5.8	16.4	50.7
National Wealth Scale(tertiles)				
1st	5.3	4.4; 6.2	8.5	243.6
2nd	6.4	4.6; 8.2	14.2	300.4
3rd	3.9	2.3; 5.6	21.4	183.1
*Brazilian Food Insecurity Scale* (EBIA)				
Food security	5.3	3.9; 6.6	12.8	380.9
Mild insecurity	5.0	3.9; 6.2	11.3	265.7
Moderate or severe insecurity	5.6	3.4; 7.8	19.9	80.3
Sewage				
General network	5.6	4.4; 6.8	10.7	580.7
Other	4.1	3.0; 5.2	13.5	146.3
Child age (years)				
< 2	6.6	5.2; 8.0	10.8	369.8
≥ 2	4.3	3.2; 5.3	12.7	357.2
Child sex				
Male	5.6	4.4; 6.8	11.0	394.4
Female	4.9	3.6; 6.2	13.5	332.6
Child race/skin color				
White	4.8	3.8; 5.9	11.4	278.9
Mixed-race	5.3	4.0; 6.6	12.9	381.5
Black	6.7	3.1; 10.4	27.5	60.5
Maternal educational level (completed years)				
0-7	7.3	5.1; 9.5	15.1	216.9
8-10	5.2	3.7; 6.8	14.6	155.0
11	4.7	3.3; 6.1	15.0	263.1
≥ 12	3.8	2.3; 5.4	20.7	91.9
Maternal age (years)				
< 20	3.9	1.7; 6.1	28.7	36.2
20-29	4.2	3.3; 5.2	11.5	290.3
30-39	6.2	4.9; 7.4	10.7	320.3
≥ 40	8.9	4.0; 13.7	27.7	80.2
Mother lives with partner				
Yes	5.3	4.3; 6.3	9.6	547.6
No	4.9	3.2; 6.6	17.8	179.4

95%CI: 95% confidence interval; CV: coefficient of variation.

* Mothers overweight was classified using body mass index (BMI)-for-age Z-score (BAZ) > 1 (adolescents, age < 20 years old) or BMI ≥ 25.0kg/m^2^ (adults); Undernourished child: stunting (height-for-age Z-score - HAZ < -2), wasting (BAZ < -2 (age ≥ 2 years old) or weight-for-height Z-score (WHZ) < -2 (age < 2 years old), or underweight (weight-for-age Z-score - WAZ < -2);

** Mother-child dyads (x 1,000), the cell value x 1,000 represents the total of mother-child dyads estimate in population with the condition.

In 2006, 41.6% of mothers and 8.7% of children were overweight (using WHZ or BAZ), 8.4% of children were stunted, and 4.2% of mothers and 1.8% of children were underweight. The prevalence of overweight in the mother-child dyad was 3.1% using WHZ and 5.2% using WHZ or BAZ. The prevalence of undernourishment in the mother-child dyad was 0.1%, and 2.7% of the mother-child dyad were classified as having the overweight mother and undernourished child (data not shown in tables).

**Figure 1:**
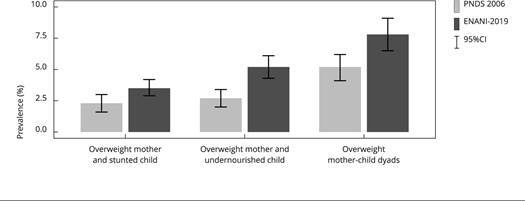
Prevalence (%) of malnutrition among mother-child dyads in Brazil in 2006 and 2019.

Source: *Brazilian National Survey on Child Nutrition* (ENANI-2019) and the *Brazilian National Survey of Demography and Health of Women and Children* (PNDS 2006) ^15^. Notes: mothers’ overweight was classified using body mass index (BMI)-for-age Z-score (BAZ) > 1 (adolescents, age < 20 years old) or BMI ≥ 25.0kg/m^2^ (adults); Stunting classification criteria: height-for-age Z-score (HAZ) < -2; Undernourished child criteria: stunting (HAZ < 2), wasting (BAZ < -2 (age ≥ 2 years old) or weight-for-height Z-score (WHZ) < -2 (age < 2 years old), or underweight (weight-for-age Z-score - WAZ < -2).

The prevalence of malnutrition in mother-child dyads increased from 2006 to 2019. The prevalence of dyads classified as overweight mothers and undernourished children increased from 2.7% to 5.2%, which is an increase of 92% (2.5 percentage points) from 2006 to 2019. The prevalence of overweight in mother-child dyads increased 50% (2.6 percentage points) in the same period. The prevalence of overweight mothers and stunted children increased from 2006 to 2019, but the difference was not statistically significant (Figure 1). Similar results were found when the analyses of the ENANI-2019 data were restricted to the mother’s age group of PNDS 2006 (data not shown in tables).

## Discussion

This study has three main findings. First, overweight in mother-child dyads and overweight in the mother and any form of undernourishment in the child (double burden of malnutrition) are the primary expressions of malnutrition at the household level in Brazil, affecting over 1.8 million dyads. Second, overweight was more prevalent in the Southern Region of the country and the double burden of malnutrition was also higher in dyads with lower maternal education level and older age and in young children (< 2 years old), although for double burden of malnutrition, the differences were not statistically significant. Third, malnutrition in mother-child dyads had a steep increase from 2006 to 2019, in which overweight in mother-child dyads increased by 50% and double burden of malnutrition increased by 92%.

In Brazil, overweight affected over one million mother-child dyads in 2019. Mother and child share genetics and sociodemographic, contextual, and behavioral characteristics. Parents have a substantial impact on children’s health behaviors ^26^ and nutritional status ^27^. Regarding child feeding, the influence can be explained by child self-regulation, parental eating practices, and family meal environment ^28^. Obesogenic lifestyles and behavioral traits, including diet and physical activity, can be easily assimilated by children via family socialization ^29^ ^,^ ^30^.

A higher prevalence of overweight was observed in the dyads from the South compared to those in Central-West Brazil. The drivers of nutritional transition can partially explain this regional difference, e.g., urbanization and dietary patterns, which can differ throughout Brazil ^31^. The prevalence was also higher in children 2-5 years old than in those ≤ 2 years old. In line with these results, a systematic review and meta-analysis with data from high- and middle-income countries reported that the association between obesity in parents and children was stronger in older children than in younger children ^27^. Besides child self-regulation, eating behaviors, parental supervision, and children’s socialization ^32^, these differences may reflect the cumulative effect of more prolonged common family exposure.

Childhood overweight has short- and long-term consequences, such as metabolic changes related to cholesterol, triglycerides, glucose profiles, high blood pressure, and an increased risk of obesity later in life ^33^ ^,^ ^34^ ^,^ ^35^. Thus, public health interventions should recognize the association between maternal and child nutritional status and encourage maternal/parental healthy eating attitudes rather than only educate parents on how to feed their children ^27^.

The prevalence of double burden of malnutrition seems to be a growing problem in Brazil and may reflect the pattern of nutritional transition that has occurred in the country over time. From 2006 to 2019, the prevalence of double burden of malnutrition almost doubled. An increasing prevalence of overweight was observed in the Brazilian population, whereas underweight is under control in the adult population ^36^. The prevalence of stunting and underweight in children decreased significantly in Brazil from 1974 to 2006, and the prevalence of overweight in children < 5 years old remained constant ^36^. However, from 2006 to 2019, the prevalence of overweight and wasting increased, and stunting was stable in Brazilian children under 5 years old, but when considering the < 1-year-old group, the prevalence increased from 4.9% to 9% ^15^ ^,^ ^37^ ^,^ ^38^.

Other low- and middle-income countries have registered a decreased in underweight and stunting prevalence among children and an increase in overweight prevalence among women by approximately one percentage point per year ^1^. A study with data from Demographic and Health Surveys (DHS) in 54 low- and middle-income countries showed that overweight mothers and undernourished children ranged from 1.8% in families in Ethiopia to 15.9% in Egypt and South and Southeast Asia. There has been a steady decline in childhood undernutrition and an increasing trend toward overweight among women. This transition has been accompanied by economic growth and demographic and epidemiological transitions in recent decades. In a study carried out in Peru ^9^ using individual-level data collected in nationally representative household surveys from 1996 to 2017, the prevalence of double burden of malnutrition decreased from 10% to 7% during the period. The possible explanation may be related to the improvement in socioeconomic factors that occurred in Peru in the previous decades, which might have contributed to a change in the pattern of children with normal nutritional status having overweight mothers ^9^.

Increasing evidence show the association of increasing overweight prevalence with higher consumption of ultra-processed foods ^39^ ^,^ ^40^. This food group has been associated with a greater risk of obesity ^41^ and noncommunicable diseases ^42^ and may be related to stunting when consumed in the first 1,000 days of life ^42^. The consumption of energy-rich and nutrient-poor foods and the unequal food distribution at the household level can contribute to adult overweight and impaired child growth ^43^. Maternal education also seems to influence the prevalence of maternal overweight and child undernourishment. A study conducted using population-representative data from the DHS of Bangladesh, India, Nepal, Pakistan, Myanmar, Timor, the Maldives, and Cambodia found that the double burden of malnutrition in mother-child dyads was higher in dyads with lower maternal education and higher age ^8^.

ENANI-2019 allowed an unprecedented assessment of the combined situation of the many forms of malnutrition in mother-child dyads in Brazil. The use of individual-level data from nationally representative surveys is a strength of the study. Furthermore, ENANI-2019 and PNDS 2006 used comparable study designs and data collection methods, allowing the assessment of the trend in malnutrition over 13 years. Anthropometric measures of ENANI-2019 were collected using standardized procedures. The training was conducted in all Brazilian states by qualified instructors, with real-time eletronic monitoring of children’s anthropometric measurements ^17^. The study has some limitations, such as the inclusion of biological mothers aged 16-64 years old in ENANI-2019, which may limit the comparison with PNDS 2006. However, similar results were found when the analyses of ENANI-2019 data were restricted to the mother’s age group (14-49 years old) of PNDS 2006.

Malnutrition is a public health challenge worldwide and knowing how it affects the population is a pivotal step in planning public health intervention. ENANI-2019 assessed the combined situation of several forms of malnutrition in the Brazilian maternal-child dyad, which had not been updated since 2006. In 2019, the most frequent forms of malnutrition in the dyads were overweight in the mother and child, and overweight in the mother associated with any form of undernourishment in the child. Both problems have increased since 2006; overweight in the dyad increased from 5.2% to 7.8%, and the double burden of malnutrition increased from 2.7% to 5.2%. Public health interventions need to focus on dyads in vulnerable situations such as lower formal education and higher maternal age, considering the relationship between maternal and child nutrition status.
